# The natural history of pregnancies with a diagnosis of Trisomy 18 or Trisomy 13; a retrospective case series

**DOI:** 10.1186/1471-2393-13-209

**Published:** 2013-11-18

**Authors:** Orla A Houlihan, Keelin O’Donoghue

**Affiliations:** 1 Anu Research Centre, Department of Obstetrics and Gynaecology, University College Cork, Cork University Maternity Hospital, Wilton, Cork, Republic of Ireland

**Keywords:** Trisomy 18, Trisomy 13, Aneuploidy, Pregnancy

## Abstract

**Background:**

Trisomy 18 (T18) and trisomy 13 (T13) are the second and third commonest autosomal aneuploidy syndromes respectively. While specific aspects of affected pregnancies have been documented in the literature, few studies document the overall natural history of the trisomies. This study aimed to examine the natural history (including diagnosis, pregnancy outcome, complications and survival) of T18 and T13 pregnancies in a setting where termination of pregnancy for fetal abnormality is not available.

**Methods:**

Cases were identified using birth registers, labour ward records, annual reports, medical records, ultrasound reports and reports from prenatal genetic testing. All identified T18 and T13 pregnancies in the study region from 2001 to 2012 were included. Individual chart reviews were performed for each case. Data were analysed using SPSS Version 20.

**Results:**

Forty-six T18 and twenty-four T13 pregnancies were identified. Most T18 cases (65%) were diagnosed prenatally, while only one third (33%) of T13 cases were prenatally diagnosed. Only three T18 pregnancies and one T13 pregnancy were electively terminated. A proportion of undiagnosed infants were delivered by emergency caesarean section. 48% (T18) and 46% (T13) infants survived following birth, for a median of 1.5 days (T18) and 7 days (T13). One T13 infant is currently alive over one year of age.

**Conclusions:**

This large series provides information for professionals and women regarding the natural histories of trisomies 18 and 13. These pregnancies can go undiagnosed antenatally without routine anomaly scanning. While many fetuses die in-utero, postnatal survival is possible.

## Background

Trisomy 18 (T18) is the second commonest autosomal aneuploidy after Trisomy 21, with an approximate prevalence of 5.05 per 10,000 births in the region in which this study was conducted (Cork, Ireland). Trisomy 13 (T13) is the third commonest autosomal aneuploidy with an approximate local prevalence of 2.16 per 10,000 births [1]. In the region studied, there were 90,424 live births in total during the years 2001–2012 inclusive [2], making these aneuploidy syndromes a worthy research topic. These syndromes are associated with multisystem abnormalities, including cardiovascular, neurological, renal, gastrointestinal and skeletal malformations, which have been described in the literature [3-6].

In the published literature, more studies of T18 pregnancies are available. Most concentrate on specific aspects of the pregnancies, including risk of fetal loss [7] and survival time, while all [8] report a high risk of spontaneous fetal loss, with pregnancy loss rates ranging from 72% to 87% for T18, and between 49% and 66% for T13 pregnancies [8,9]. The majority of liveborn T18 and T13 infants (>90%) in most studies survive less than one year, with some reports noting a significantly increased survival time for T18 females when compared with males [10,11]. A large Texas-based study by Vendola et al. [12] reported a 3% one year survival rate for both T18 and T13 infants.

While few infants with trisomy 18 survive past one year of life, a review by Cereda et Carey [5] reported that approximately half of affected babies live longer than one week. A study by Baty et al. [13] reported a much greater one-year survival rate of 42% for T18 infants and 38% for T13 infants, however, this study was based on data collected from questionnaires completed by members of S.O.F.T. (Support Organisation for Trisomy 18, 13 and Related Disorders), which may have resulted in non-response bias. The median reported survival time of affected liveborn infants varies between 3 and 15 days for both trisomies [8-10,14,15]. Case reports of T18 and T13 individuals surviving for much longer periods of time have also been published: Petek et al. [16] described a nineteen year old girl with a full T18 karyotype, Smith et al. [17] reported an eleven-year-old with T18, and in a survey by Bruns et al. [6], survival times of 103.3 months were reported by mothers of 13 trisomy 18 newborns who were still alive at the time of participation in the study. Redheendran et al. [18] reported an eleven and a nineteen year old with T13 karyotypes.

The prevalence of both T18 and T13 has increased in recent years. One United Kingdom-based study [19] reported an increase in prevalence from 0.20 to 0.65 per 1,000 births for trisomy 18 and from 0.08 to 0.23 per 1,000 births for trisomy 13 between the years 1985 to 2007. This change has been attributed to increasing maternal age and increased prenatal screening for fetal aneuploidies, which is now recommended for all pregnancies by the Royal College of Obstetricians and Gynaecologists [20]. In contrast, in the Republic of Ireland, where our series is based, prenatal screening for fetal aneuploidy is not part of routine antenatal care. There is no national policy on the provision of prenatal screening for aneuploidy. In an Irish study, Lynch and Malone [21] found that while 72% of healthcare professionals surveyed believed that detailed ultrasound scans should be performed for fetal anomaly, only 10% routinely discussed prenatal screening for fetal aneuploidy. This is in spite of findings that more than 73% of Irish pregnant woman would avail of fetal anomaly scans or biochemical testing if it was made available to them [22].

Few studies report data relating to the entire natural history of affected pregnancies. The published literature on these trisomies is limited by small sample size and high rates of elective termination of pregnancy following prenatal diagnosis (up to 78%) [9]. Elective termination of pregnancy for fetal abnormality is illegal in the Republic of Ireland. While Irish women can access termination of pregnancy in other jurisdictions, the majority tend to allow pregnancies diagnosed with fetal abnormality to continue. Therefore, we have an ideal opportunity to conduct observational studies on pregnancies affected by trisomies 18 and 13.

Our aim was to study the natural history of pregnancies with a fetal or neonatal diagnosis of trisomies 18 and 13. In doing so, we aimed to provide clinicians with a better indication of the course of affected pregnancies, in order to assist with counselling and management and to improve the quality of care. Knowledge of the outcome, complications and infant survival time of previous T18 and T13 pregnancies could assist parents and obstetricians in making more informed decisions related to the management of affected pregnancies in the future.

## Methods

This was a retrospective descriptive cohort study. Ethical approval for the proposed study was granted from the Cork Research and Ethics Committee (CREC) of the Cork Teaching Hospitals in November 2011. All pregnancies and infants with a diagnosis of T18 or T13 cared for in all hospitals operating in the Cork region during the period from January 2001 to July 2012 were included. These were the Erinville Hospital (closed 2007), St. Finbarr’s maternity hospital (closed 2007), the maternity unit in the Bons Secours Hospital (closed 2007) and Cork University Maternity Hospital, which was formed from the amalgamation of all three maternity units and opened in April 2007. All of the women in the study were booked for consultant-led antenatal care at one of these units. Input from a consultant obstetrician was available at each antenatal visit for all pregnancies involved in the study and a consultant obstetrician reviewed all women with a fetal diagnosis of trisomy 18 or trisomy 13.

Cases were identified using birth registers, labour ward records, annual reports, medical records, ultrasound reports and reports from prenatal genetic testing. Identified cases were then verified using the EUROCAT (European surveillance of congenital anomalies) Cork and Kerry Registry, which records data for the region studied. Pregnancies studied all had a diagnosis of T18 or T13 confirmed by karyotype either prenatally or postnatally. Trisomies of the long (q) arm of chromosome 18 or 13 were also included. Mosaic cases were excluded from the study. Following verification, maternity charts were requested from the relevant storage facilities and individual case reviews were subsequently performed. Information was collected in relation to parental demographics, gestation at diagnosis, method of diagnosis, structural abnormalities, pregnancy complications (maternal and fetal), pregnancy outcome, timing of fetal or neonatal death, mode of delivery, delivery complications and neonatal survival time.

All data collected was anonymised and entered into a Microsoft® Excel® 2010 spread sheet, saved onto a password-protected secure external hard drive. The data was then imported into SPSS® Statistics Version 20 and was coded appropriately in order to enable compatibility with SPSS. An assessment of normality was performed for certain variables in the dataset, using histograms, normal Q-Q plots, detrended Q-Q plots and the Shapiro-Wilk est. Descriptive statistics were used to present the data. Continuous variables were summarised as medians and interquartile ranges (IQR) (nonparametric data). Categorical variables were summarised as percentages. The Chi-Squared goodness of fit test, Mann–Whitney U test and exact hypothesis test with binomial distribution were performed to analyse data. Statistical significance was reported when a two-sided P value was found to be less than or equal to 0.05.

## Results

### Fetal characteristics

Forty-six cases of T18 and twenty-four cases of T13 were identified. One T13 infant had an unbalanced Robertsonian translocation t (13; 14), in which three copies of the long arm of chromosome 13 were detected. The remainder of the cases were full trisomy karyotypes. One T18 mosaic case was identified; however, this was subsequently excluded from the series as two thirds of the infant’s cells were found not to contain the trisomy, and the infant did not demonstrate a characteristic T18 phenotype. Two T18 infants were part of twin gestations; one dichorionic diamniotic twin pregnancy and the other a monochorionic diamniotic pregnancy. One T13 infant was part of a dichorionic diamniotic twin pregnancy. In all twin pregnancies, the other twin had a normal karyotype and had an uncomplicated delivery.

Gender distribution for the T18 and T13 groups is shown in Table 1. There was no significant difference between the numbers of overall or liveborn T18 male and female cases, although 75% more females than males were present in the liveborn group. Twice as many male than female T13 cases were identified, with the male to female ratio rising to 9:2 when only those fetuses that were born alive were considered. The greater number of male than female T13 liveborn infants was not found to be statistically significant (p = 0.065).

**Table 1:** Gender distribution for the T18 and T13 cohort

	**Male**	**Female**	**P-value**
Overall T18	21	24	0.655
Live born T18	8	14	0.201
Overall T13	14	7	0.127
Live born T13	9	2	0.065

Gender distribution for the T18 and T13 cohort.

### Parental characteristics

Thirty seven per cent (17/46) of women carrying T18 fetuses and thirty three per cent (8/24) of those carrying T13 fetuses had a history of a previous miscarriage. The median parity for mothers of both trisomies was one (T18 IQR 0–2; T13 IQR 0–2). The median maternal age at delivery was 38 years (IQR 34–40 years) for T18 pregnancies and 35 years (IQR 28.5-38 years) for T13 pregnancies. The proportion of mothers over 35 years of age was 74% for T18 and 54% for T13, which was significantly higher for both trisomies than the 28% for all maternities in the same region [2]. (T18: X2 (1, n = 46) = 51.364, p < 0.001) (T13: X2 (1, n = 24) = 8.987, p = 0.03). No parents had previously conceived a T18 or T13 fetus, however, one 39-year old T18 mother had a trisomy 21 pregnancy the previous year. Six parents of T18 fetuses and two of T13 fetuses later underwent karyotyping. All were subsequently found to have normal karyotypes, including the parents of the T13 fetus with the t (13; 14) translocation and the parents who had conceived both a trisomy 18 and a trisomy 21 fetus.

### Diagnosis of trisomy

Sixty five per cent (30/46) of T18 cases were prenatally diagnosed, in comparison with only thirty three per cent (7/21) of T13 cases. Three T13 cases that ended in miscarriage in the first trimester were excluded from these figures, as they died in-utero before an ultrasound scan was performed. The median gestation at diagnosis was 22 weeks for T18 (IQR 15–31.5 weeks) and 20.5 weeks for T13 (IQR 15.5-28 weeks). The most common reasons for invasive diagnostic testing were abnormalities detected on routine ultrasound scans, polyhydramnios and small for gestational age fetuses. The commonest fetal abnormality detected on ultrasound scans was increased nuchal translucency (nine T18 cases; three T13 cases). None of the women studied underwent first or second trimester prenatal screening for fetal aneuploidy. Following the opening of Cork University Maternity Hospital rates of prenatal diagnosis increased from 42.9% to 87.5% for T18 and from 16.67% to 61.5% for T13. Using Fisher’s exact probability test, this increase in prenatal diagnosis was found to be statistically significant for the T18 cases (p = 0.048). While the rate of prenatal diagnosis for T13 fetuses increased by almost 45% in the period following the opening of CUMH, this was not found to be statistically significant (p = 0.354). Of the 17 T18 pregnancies in which a detailed structural anomaly scan was performed, 14 (82%) T18 fetuses were antenatally diagnosed. Six structural anomaly scans were performed during T13 pregnancies and three (50%) of these fetuses were prenatally diagnosed.

### Congenital defects

The congenital defects reported for the T18 and T13 cases are shown in Table 2. For the T18 fetuses, the most common defects reported were ventricular septal defect (16/46; 35%) and abnormal posturing of the hands (9/46; 20%) or feet (10/46; 22%). The commonest abnormalities identified in T13 fetuses were a cleft lip and/or palate (11/24; 46%), holoprosencephaly (7/24; 29%) and talipes or rocker bottom feet (6/24; 25%). A detailed structural anomaly scan was carried out in seventeen T18 (17/46; 37%) and six T13 (6/24; 25%) pregnancies to further investigate abnormalities detected on routine ultrasound scans, most commonly increased nuchal translucency (2 T18) and intrauterine fetal growth restriction (16 T18; 3 T13). The structural anomalies specific to those pregnancies in which a detailed anomaly scan was performed are shown in Table 3. Post mortems were only carried out in two T18 (2/46; 4%) and two T13 cases (2/24; 8%).

**Table 2:** Structural abnormalities for T18 and T13 cohorts

	**Trisomy 18 (N = 46)**	**Trisomy 13 (N = 24)**
**Cardiac**		
Ventricular septal defect	16 (35%)	5 (21%)
Transposition of Great Arteries	1 (2%)	2 (8%)
Patent ductus arteriosus	1 (2%)	1 (4%)
**Cerebral**		
Holoprosencephaly	0	7 (29%)
Cerebral ventriculomegaly	4 (9%)	3 (13%)
Microcephaly	1 (2%)	3 (13%)
Dandy Walker variant	1 (2%)	2 (8%)
**Structural**		
Cleft lip/palate	4 (9%)	11 (46%)
Talipes/rockerbottom feet	10 (22%)	6 (25%)
Clenched hands	9 (20%)	1 (4%)
Polydactyly	0	5 (21%)
Low set ears	2 (4%)	4 (17%)
Short long bones	4 (9%)	0
Overlapping fingers	3 (7%)	1 (4%)
Micrognathia	3 (7%)	0
Ocular hypotelorism	1 (2%)	2 (8%)
Syndactyly	2 (4%)	0
Clinodactyly	1 (2%)	1 (4%)
Prominent forehead	1 (2%)	1 (4%)
**Gastrointestinal**		
Small/absent stomach	6 (13%)	2 (8%)
Omphalocele	6 (13%)	0
Echogenic bowel	1 (2%)	3 (13%)
Umbilical herniation	1 (2%)	1 (4%)
**Respiratory**		
Pleural effusion	4 (9%)	0
Diaphragmatic hernia	4 (9%)	0
**Renal**		
Enlarged/cystic kidneys	4 (9%)	5 (21%)

Structural abnormalities for T18 and T13 cohorts.

**Table 3:** Structural abnormalities for T18 and T13 cohorts who nderwent detailed anomaly scans

	**Trisomy 18 (N = 17)**	**Trisomy 13 (N = 6)**
**Cardiac**		
Ventricular septal defect	11	1
Transposition of Great Arteries	1	0
Patent ductus arteriosus	0	1
**Cerebral**		
Holoprosencephaly	0	2
Cerebral ventriculomegaly	2	0
Microcephaly	1	1
Dandy Walker variant	1	1
**Structural**		
Cleft lip/palate	2	4
Talipes/rockerbottom feet	5	2
Clenched hands	5	1
Polydactyly	0	1
Low set ears	1	0
Short long bones	3	0
Overlapping fingers	0	1
Micrognathia	2	0
Ocular hypotelorism	1	1
Syndactyly	2	0
**Gastrointestinal**		
Small/absent stomach	3	1
Omphalocele	4	0
Umbilical herniation	1	0
**Respiratory**		
Pleural effusion	1	0
Diaphragmatic hernia	3	0
**Renal**		
Enlarged/cystic kidneys	1	3

Structural abnormalities for T18 and T13 cohorts who underwent detailed anomaly scans.

### Pregnancy outcome

**Figure 1:**
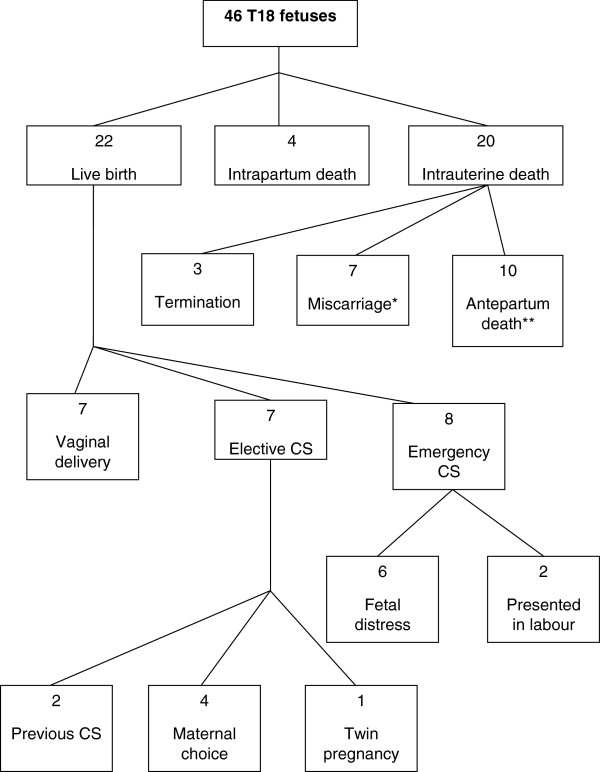
**T18: Pregnancy outcome, mode of delivery (live born cases) and indications for caesarean section.** *Miscarriage: Fetal death in-utero before twenty weeks gestation. **Antepartum death: Fetal death in-utero at or after twenty weeks gestation.

**Figure 2:**
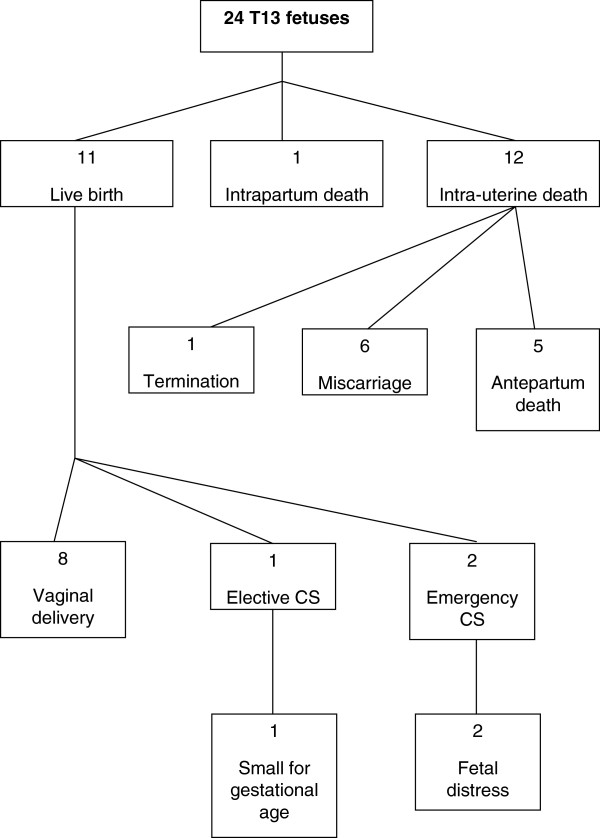
T13: Pregnancy outcome, mode of delivery (live born cases) and indications for caesarean section.

The outcome for the T18 and T13 pregnancies is shown in Figure 1 and Figure 2 respectively. Almost half of T18 (22/46; 48%) and T13 (11/24; 46%) pregnancies resulted in the delivery of a liveborn infant. Of note, only three T18 cases (6%) and one T13 case (4%) were electively terminated.

### Pregnancy complications

Thirty-five T18 fetuses (35/46; 76%) and twelve T13 fetuses (12/24; 50%) were noted to be small for gestational age. The most common pregnancy complication identified was polyhydramnios, which was present in thirteen T18 pregnancies (13/46; 28%) and three T13 pregnancies (3/20; 15%) in the second and third trimesters. Antepartum haemorrhage occurred in eight T18 (8/46; 17%) pregnancies and four T13 (4/24; 17%) pregnancies; all were of a small quantity and resolved spontaneously. Pre-eclampsia was reported in two T18 (2/36; 6%) and two T13 (2/17; 12%) pregnancies. The deliveries of the liveborn T18 and T13 fetuses were largely uncomplicated. One T18 mother developed an acute stress reaction requiring psychiatric intervention following the birth of her baby, whose trisomy was not antenatally diagnosed. One T13 mother developed endometritis following delivery.

### Gestation at delivery

The median gestational age at delivery was 36.5 weeks (IQR 33.75 – 40 weeks) for T18 liveborn infants and 38 weeks (IQR 35–40 weeks) for T13 liveborn infants. Eleven (50%) T18 infants and 2 (18%) T13 infants were delivered preterm. The most common reason for preterm delivery was obstetric intervention because of concerns regarding fetal wellbeing and poor growth detected on ultrasound scans in undiagnosed fetuses (5 T18 cases; 2 T13 cases). The other T18 fetuses that were delivered prematurely either underwent spontaneous labour at an early gestation (4 cases), or the fetus was prenatally diagnosed and the decision to deliver prematurely was made by the involved obstetrician in each case (3 cases).

### Mode of delivery

The mode of delivery for the liveborn T18 infants is shown in Figure 1. A high proportion of cases were delivered by caesarean section; eight infants (8/22; 36%) were delivered by emergency caesarean section and seven infants (7/22; 32%) by elective caesarean section. The mode of delivery for the liveborn T13 infants is shown in Figure 2. While the majority (8/11; 73%) were born by normal vaginal delivery, two cases (2/11; 18%) were delivered by emergency caesarean section and one case (1/11; 9%) by elective caesarean section. The most common indication for emergency caesarean section in both groups was distress in an undiagnosed fetus, as reported in six liveborn T18 infants (6/22; 27%) and two liveborn (2/11; 18%) T13 infants.

### Survival time

**Figure 3:**
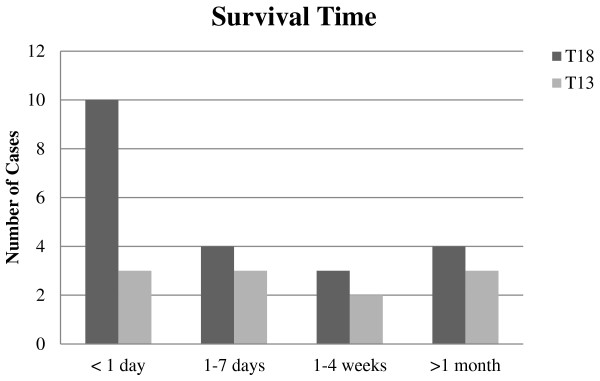
**Survival time for liveborn T18 and T13 cases.** The definite survival time for one T18 liveborn infant is unknown and therefore unable to be represented in bar chart.

The survival time for live born T18 and T13 infants is shown in Figure 3. The 22 T18 live born infants survived for a median time of 1.5 days (range; one hour-sixteen weeks). One female T18 infant (born 2001) had an unknown duration of survival but it is highly likely that she died in the early neonatal period as no hospital record was found for her after birth. The female T18 liveborn infants survived for a longer period than the male liveborn infants. This was found to be statistically significant using the Mann–Whitney U test (p = 0.016). The median survival for the eleven liveborn T13 infants was seven days (range; fifteen minutes- over one year). Of note, one male T13 liveborn infant (born October 2011) is still alive at two years of age. No T18 or T13 liveborn received invasive ventilation or cardiovascular support following birth. “Comfort care” was practiced, and the decision was made not to perform fetal monitoring during labour for 4 T18 and 2 T13 liveborn infants.

## Discussion

### Main findings

This study reports all identified T18 and T13 pregnancies in the Cork region over an 11-year period from January 2001 to July 2012. Our cohort of forty six T18 pregnancies and twenty-four T13 pregnancies is one of the largest recent case series available. Our study identified 46 T18 (47 + 18) and 23 T13 (47 + 13) cases diagnosed between January 2001 and June 2012, in addition to one unbalanced Robertsonian translocation (46 t(13; 14)) who demonstrated the characteristic phenotypical features of a T13 karyotype. The median maternal age of both trisomy cohorts was significantly older than the median maternal age of the general population. Only three T18 and one T13 pregnancy were electively terminated after diagnosis. The median gestation at diagnosis for both trisomies was in the second trimester (T18 22 weeks; T13 20.5 weeks) and the most common indications for invasive diagnostic testing were abnormalities detected on ultrasound scans, polyhydramnios and concern with regard to fetal growth. The rate of diagnosis for both T18 and T13 increased following the opening of the local large tertiary referral hospital. Congenital defects in multiple organ systems were reported for both trisomies, however the percentage of fetuses affected was lower than that reported in other studies [11,23]. This is likely due to the fact that detailed second-trimester structural anomaly scans were performed in only seventeen (37%) T18 and six (25%) T13 pregnancies.

Eleven T18 liveborn infants and two (18%) T13 liveborn infants were delivered preterm, most commonly as a result of obstetric intervention due to concerns relating to fetal wellbeing. A large proportion of T18 and a smaller proportion of T13 infants were delivered by emergency caesarean section, the most common indication for which was concern for fetal wellbeing in undiagnosed pregnancies. The majority of pregnancies and births for both trisomies were uncomplicated, with the most common pregnancy complication being polyhydramnios. While many T18 and T13 cases died spontaneously in-utero, almost half of cases in both trisomy cohorts survived for some time following birth. In line with other reports, we found that most liveborn T18 and T13 fetuses survived for a very short period of time, with T18 female infants surviving for a longer period of time than T18 male infants; however, one T13 infant from the series is still alive at (currently) two years of age.

### Strengths

This research is strengthened by the fact that all cases included in the study were definitively diagnosed by means of fetal karyotyping either prenatally or postnatally. Permission was granted to examine the birth records of all maternity hospitals caring for births in the region during the time period specified. Also, the relevant pregnancy chart was obtained for each case identified. In addition, each case was verified using the EUROCAT database. This contributed to the inclusiveness and completeness of the study. The low rate of elective termination of pregnancy for both trisomies (T18 6%; T13 4%) also strengthens the study, as the vast majority of pregnant women chose to continue with affected pregnancies. This is in contrast to studies that report high rates of elective termination of pregnancy [9].

### Limitations

The main limitation of this study is its small sample size, which is related to the low prevalence of the trisomies. Our sample size, however, is larger than that in many previously published studies. Our study also relied on accurate documentation of the course of affected pregnancies in written medical records. It is difficult to be certain that all of the information recorded in each chart is completely accurate, especially if some was written retrospectively resulting in recall bias.

### Interpretation

This information relating to the natural history of T18 and T13 pregnancies should be of benefit internationally. It is important that, on receiving a fetal diagnosis of T18 or T13, parents are assisted to make an informed decision when determining whether to continue with the pregnancy or access termination of pregnancy. While many parents will chose the latter option, detailed knowledge of the likely course of the pregnancy is helpful to those who chose to continue.

In the Republic of Ireland, prenatal screening for fetal aneuploidy syndromes is not routinely offered, and nationally around 50% of women are offered a detailed second trimester anomaly ultrasound scan. The lack of prenatal screening and structural anomaly scans likely contributed to the relatively high number of T18 and T13 fetuses in this study who were not diagnosed until after birth. A prenatal diagnosis of a T18 or T13 fetus could enable clinicians to appropriately counsel parents antenatally and to prepare them for the likely outcome. An unexpected postnatal diagnosis where there has been anticipation of a normal outcome can be extremely traumatic for parents, who may then have little time to adjust to the reality of an infant with significant malformations and a high risk of neonatal death. In our study, one pregnant woman who discovered following birth that her baby had T18 subsequently suffered an acute stress reaction requiring psychiatric intervention. Prenatal anomaly screening and diagnosis has been reported as a practice of which the majority of both healthcare professionals and pregnant women are in favour [21,22].

An important finding in this study is the high rate of caesarean section performed for T18 and T13 fetuses, particularly the high rate of emergency deliveries. As the majority of fetuses who were delivered by emergency caesarean section had not been prenatally diagnosed, the question must be raised as to whether obstetricians and mothers would have chosen this mode of delivery if they had been aware that the fetus had a lethal aneuploidy syndrome. Had routine screening for fetal abnormalities been offered to pregnant women, the antenatal diagnosis of T18 or T13 may have influenced the delivery method chosen for the affected fetuses. The increased risks of emergency caesarean section compared with other modes of delivery are well documented in the literature, as are the potential implications of undergoing an emergency caesarean section for subsequent pregnancies [24].

## Conclusion

This study represents one of the largest recent case series detailing the natural history of T18 and T13 pregnancies. Due to the increasing prevalence of these aneuploidy syndromes and their relative rarity, these reports are necessary to provide healthcare professionals with an indication of the potential course of the pregnancies, and to facilitate appropriate parental counseling. As many published studies of the natural history of T18 and T13 pregnancies are limited by small sample sizes, a meta-analysis of previously published data could be of benefit in increasing the statistical power of findings. Also, specific clinical guidelines for the management of pregnancies with a fetal diagnosis of T18 and T13, detailing the potential outcomes and complications of these pregnancies, in addition to the likelihood of each of these occurring, would be beneficial for healthcare professionals caring for affected pregnancies.

### Details of ethical approval

Expedited ethical approval for the proposed study was granted from the Cork Research and Ethics Committee (CREC) of the Cork Teaching Hospitals in November 2011.

## Abbreviations

T18: Trisomy 18; T13: Trisomy 13; IQR: Interquartile range.

## Competing interests

The authors declare that they have no competing interests.

## Authors’ contributions

KOD designed the study and assisted with data collection, analysis and writing the paper. OAH identified the cases, collected and analysed the data and wrote the paper. Both authors read and approved the final manuscript.

## Pre-publication history

The pre-publication history for this paper can be accessed here:

http://www.biomedcentral.com/1471-2393/13/209/prepub
